# Optimization of wind-solar-gas-storage integrated energy systems under the carbon and green certificate markets

**DOI:** 10.1371/journal.pone.0346530

**Published:** 2026-04-24

**Authors:** Debin Fang, Heng Zhou, Pengyu Wang, Zeyu Xing

**Affiliations:** 1 Research Center for Complexity Science and Management, Wuhan University, Wuhan, China; 2 State Key Laboratory of Water Resources Engineering and Management, Wuhan University, Wuhan, China; 3 South-Central Minzu University, Wuhan, China; Van Lang University: Truong Dai hoc Van Lang, VIET NAM

## Abstract

This study investigates the optimization of wind-solar-gas-storage integrated energy systems (IES) under the dual-market mechanisms of carbon emission trading (CET) and green certificate trading (GCT). A unified analytical framework is developed to examine the techno-economic and environmental performance of IES within these combined market structures. The results indicate that the concurrent implementation of CET and GCT leads to a significant reduction in operational costs while simultaneously enhancing system sustainability. Through a series of scenario analyses, we explore the impact of price fluctuations in carbon and green certificate markets on system performance and compliance expenditures. Our findings indicate that the coordinated implementation of CET and GCT can significantly reduce the operating cost of integrated energy systems—by as much as 50.2%—while also effectively lowering carbon emissions by approximately 29.9%. An increase in the carbon price yields dual benefits of cost reduction and emission abatement, with a clearly identifiable economically efficient range between 0.05 and 0.20 CNY/kg. Although higher green certificate prices can further incentivize low-carbon operation, their marginal benefits tend to diminish beyond 80 CNY per certificate. These insights underscore the importance of integrated policy frameworks that leverage the synergistic effects of CET and GCT to optimize energy system performance. The study provides valuable guidance for policymakers, system operators, and stakeholders in the energy sector, highlighting the potential for strategic investments in these market mechanisms to achieve significant environmental and economic benefits.

## 1 Introduction

The global energy transition is at a critical juncture, urgently requiring innovative solutions to reconcile the multiple demands of energy security, economic viability, and environmental sustainability [1]. IES, through the coordinated integration of energy sources, grids, loads, and storage, facilitate multi-energy complementarity by aligning diverse energy supplies (e.g., electricity, heat, cooling, and gas) with heterogeneous end-use demands. This holistic integration model significantly enhances energy efficiency and operational flexibility, improves the system’s capacity to absorb intermittent renewable energy, and thereby increases carbon reduction potential [2]. By optimizing both supply and demand sides, IES support secure and reliable energy provision and low-carbon development simultaneously—achieved through reduced fossil fuel consumption, increased shares of renewables, and responsive demand-side management [3]. As a key direction for future energy systems, IES are expected to drive the emergence of new technological pathways and business models, thereby accelerating the decarbonization and structural upgrading of the energy sector.

Alongside technological innovation, policy-driven market mechanisms play a critical role in facilitating the low-carbon transition. Among them, CET and GCT are two widely adopted market-based instruments. CET assigns a price to greenhouse gas emissions, internalizing environmental externalities and incentivizing emitters to reduce carbon emissions in the most cost-effective manner [4–6]. In contrast, GCT attributes economic value to the environmental benefits of clean electricity, offering additional revenue streams and incentives for renewable energy generation [7,8]. These two mechanisms operate from complementary perspectives—CET imposes emission constraints, while GCT rewards clean production—jointly providing economic momentum for decarbonizing energy systems. On one hand, emission caps and carbon pricing under CET increase the cost of high-emission energy sources, thereby enhancing the relative competitiveness of low-carbon alternatives [9–11]. On the other hand, green certificates serve as tradable proof of the environmental benefits of renewable electricity, encouraging generators to invest in clean energy and consumers to procure green power, creating market-driven demand for low-carbon electricity [12,13]. For example, the European Union Emissions Trading System (EU ETS), when combined with the Guarantees of Origin (GO) scheme, has led to a 43% reduction in greenhouse gas emissions and a doubling of the renewable energy share since its inception in 2005 [14–16]. Similarly, California’s cap-and-trade program and Renewable Portfolio Standard (RPS) have driven a 35% reduction in electricity sector emissions since 2013 [17]. By jointly deploying CET and GCT, policymakers can simultaneously target emission abatement costs and renewable energy incentives, accelerating the transition toward a cleaner energy structure.

However, as CET and GCT increasingly become mainstream policy instruments, the operational optimization of IES faces growing misalignments between institutional frameworks and market mechanisms—highlighting the urgent need for systemic restructuring. Existing CET and GCT schemes are primarily designed for single-energy chains and thus fail to capture the multi-energy coupling characteristics of IES. This leads to three prominent challenges in terms of policy applicability and operational efficiency: First, IES simultaneously integrates electricity, heating, cooling, and other energy forms, making it difficult to clearly attribute carbon emissions. This challenge is further complicated when intermediate processes such as dispatching and energy storage occur between generation and consumption, increasing the complexity of carbon accounting and ownership delineation. Second, while CET and GCT are designed to incentivize emission reduction and clean production, respectively, the synergistic benefits of IES—such as combined heat and power (CHP), peak shaving, and source-load coordination—are often inadequately measured or monetized. These coordination mechanisms are typically excluded from current carbon credit or green certificate valuation systems, resulting in under-recognition of IES advantages. Finally, in the context of coexisting but uncoordinated market mechanisms, IES operational decisions are subject to conflicting signals from multiple markets (e.g., electricity prices, carbon prices, certificate values), which can distort decision-making and hinder the realization of system-wide optimal outcomes.

Although extensive research has been conducted on key technologies such as IES modeling, multi-energy dispatch, energy storage optimization, and demand response, significantly improving system efficiency and energy performance, several critical research gaps remain: First, most existing studies treat energy prices, carbon costs, and green certificate incentives as exogenous variables, overlooking the feedback coupling between policy mechanisms and system behavior. This leads to limitations in policy adaptability and practical applicability of the models. Second, current research predominantly focuses on the individual impacts of CET or GCT. For instance, CET has been shown to reduce coal dependence in power systems by 12–18% [18], while GCT can increase the penetration of wind and solar power to 40% by 2030 [19]. However, these analyses often assume static carbon or certificate prices, neglecting their real-world volatility—such as a 58% fluctuation in China’s carbon market and 72% regional variation in green certificate prices in 2023 [20,21]. Finally, most optimization models adopt cost minimization as their primary objective, failing to incorporate multidimensional performance indicators such as carbon intensity, revenue stability, and system resilience. As a result, they fall short in revealing the potential synergies of integrated CET–GCT mechanisms for multi-objective coordination in IES operations [22].

To address the disconnect between institutional constraints and operational optimization in existing research, this study develops a unified analytical framework that explicitly incorporates the institutional variables and dynamic pricing characteristics of CET and GCT. The framework systematically captures the interaction mechanisms between these policies and their impacts on the energy structure, economic performance, and carbon reduction potential of wind–solar–gas–storage IES. Rather than focusing solely on operational efficiency, this study emphasizes the coordinated balance between policy responsiveness and system resilience, aiming to provide both theoretical foundations and practical guidance for the synergistic design of market mechanisms and the high-quality development of IES. Specifically, grounded in the well-established policy foundations of CET and GCT, this research addresses the following three key questions: First, how do CET and GCT interactively shape the techno-economic performance of IES under varying resource and price scenarios? Second, what synergistic effects emerge when combining tiered carbon pricing with dynamic certificate trading, and how do they reconfigure optimal energy portfolios? Third, how do cross-market price fluctuations between carbon and green certificates impact system resilience and investment priorities? To address these questions, we develop a two-stage stochastic-robust optimization framework that integrates Monte Carlo price forecasting and Conditional Value-at-Risk (CVaR) metrics, capturing both market volatility and multi-energy dispatch constraints.

**Fig 1:**
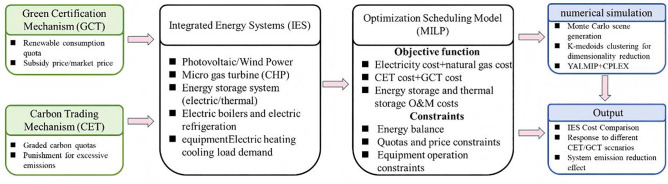
The research framework diagram.

Therefore, we propose a two-stage stochastic–robust optimization framework that integrates Monte Carlo-based price forecasting with the CVaR metric, enabling simultaneous modeling of market volatility and the multi-energy coupling constraints of IES. In the first stage, the model captures future uncertainties in CET and GCT markets by generating a set of market scenarios through Monte Carlo simulations based on historical carbon and certificate price data, and estimating their distributional characteristics. In the second stage, system operation is optimized under the given scenario set, aiming to minimize the expected total cost and CVaR-based risk exposure. The optimization objective accounts for electricity and natural gas procurement costs, carbon emission expenses, green certificate trading expenditures, and the operational costs of energy storage and combined cooling, heating, and power (CCHP) systems. To ensure compliance with existing policy frameworks, the model incorporates a tiered carbon quota pricing mechanism and green certificate quota assessment logic, thereby aligning the operational strategies with current regulatory boundaries (The research framework diagram of this article is shown in Fig 1).

This study contributes to the fields of energy policy and system integration in the following three key aspects:

First, we establish a mechanistic understanding of policy synergy in decarbonization governance by demonstrating that coordinated CET and GCT mechanisms exhibit non-linear interaction effects. The integrated framework resolves critical flexibility paradoxes inherent in renewable-dominated energy transitions, achieving enhanced environmental performance without compromising economic efficiency – a theoretical breakthrough addressing the longstanding cost-reliability trade-off in energy system optimization literature.
Second, we pioneer a tripartite optimization paradigm that operationalizes sustainable energy transitions through a novel stochastic decision-making model. This methodological innovation systematically reconciles the energy trilemma (affordability, reliability, sustainability) under market uncertainty, extending conventional single-objective energy system models through dynamic multi-criteria equilibrium analysis. The framework’s adaptive price-response architecture provides a replicable computational foundation for analyzing policy interactions in volatile energy markets.
Third, the research develops a policy calibration framework that redefines market-based climate governance boundaries. Through rigorous scenario analysis, we derive optimal CET-GCT coupling configurations that maximize emission abatement potential while maintaining market stability thresholds. These findings advance energy policy design theory by establishing principles for cross-market mechanism alignment, offering a transferable governance model for jurisdictions pursuing synergistic market instrument integration. The study further contributes to energy economics discourse by elucidating the paradigm-shifting potential of coordinated environmental markets in accelerating renewable technology diffusion and infrastructure transformation.

Collectively, these contributions reshape academic understanding of multi-instrument climate policy integration, bridging critical gaps between energy system optimization theory and market-based environmental governance practice. The proposed frameworks extend the conceptual boundaries of sustainable transition modeling while providing methodological foundations for cross-disciplinary research at the energy-economics-policy interface.

By rigorously modeling the CET-GCT nexus, this work redefines the role of market mechanisms in accelerating the clean energy transition, offering a replicable blueprint for global policymakers. The rest of this study is organized as follows. Section 2 reviews the relevant literature on IES, CET, and GCT. Section 3 presents the institutional mechanisms of CET and GCT. Section 4 introduces the structural framework of the wind–solar–gas–storage IES. Section 5 establishes the economic operation optimization model of IES under the influence of CET and GCT. Section 6 provides the case analysis, and Section 7 concludes the study.

## 2. Literature review

Current research on IES primarily focuses on achieving multi-energy coordination, improving overall energy utilization efficiency, and supporting the low-carbon transformation of energy systems under the “dual carbon” goals [23]. The development of IES is not only a natural outcome of technological advancements in the energy sector, but also a response to increasingly stringent carbon reduction constraints and the growing diversification of energy structures [24]. As a highly integrated energy management and operation paradigm, IES couples multiple energy carriers—such as electricity, natural gas, thermal energy, hydrogen, and transportation fuels—to break down the traditional silos of isolated energy systems [25]. This integration enables dynamic coordination among generation, grid, load, and storage, thereby enhancing operational flexibility, reducing overall energy consumption, and improving the accessibility and dispatchability of renewable energy sources [26]. However, the large-scale integration of renewable energy—particularly wind and photovoltaic power, characterized by volatility and uncertainty—introduces unprecedented challenges in system modeling, economic dispatch, and optimization. These challenges are especially prominent in coordinating energy flows across temporal and spatial scales, describing the coupling characteristics of heterogeneous devices, and developing real-time response mechanisms [27].

To address these challenges, researchers have explored multiple technological pathways. On the modeling side, multi-scale spatiotemporal physical modeling and optimization frameworks have been proposed to more accurately capture the dynamic evolution of multi-energy flows within IES [28]. In terms of operational mechanism design, game-theoretic approaches have been developed to characterize the interactions and strategic behaviors among different stakeholders in multi-energy systems—such as generators, loads, storage units, and end-users—thus enabling fair and efficient resource allocation [29]. In addition, with advancements in data acquisition and intelligent algorithms, data-driven methods for load forecasting and operational optimization have been widely applied in IES to enhance the system’s responsiveness to uncertainty and its adaptive control capabilities [30]. Building on these developments, growing attention has been paid to the critical role of energy storage systems in mitigating the volatility of variable renewable energy (VRE) and facilitating peak shaving and valley filling [31]. From a market mechanism perspective, researchers have also examined the impact of regional electricity market coupling in promoting cross-regional energy coordination [32]. Furthermore, to improve the efficiency of energy systems in edge areas, energy efficiency modeling methods have been proposed that account for geographical and resource heterogeneity [33]. Complementarily, the introduction of multi-community interaction mechanisms has been shown to strengthen the robustness and economic performance of IES, providing strong support for the development of resilient and self-organizing next-generation energy systems [34].

In the field of state estimation and operational decision-making for IES, the integrated application of optimization algorithms and machine learning techniques has been extensively explored [35]. At the same time, increasing attention has been directed toward the control optimization of wind power systems—particularly those based on Doubly-Fed Induction Generators (DFIGs). Due to the inherent intermittency and unpredictability of wind energy, conventional control methods often struggle to maintain system stability and power quality under high penetration scenarios. To address these limitations, researchers have proposed neural network-based Direct Power Control (NC-DPC) strategies. By incorporating artificial intelligence, these methods enable real-time modeling and adaptive control of the nonlinear dynamic behavior of wind power systems, effectively reducing power fluctuations, suppressing harmonics, and significantly enhancing dynamic response performance [36]. Building on this foundation, several advanced high-performance control strategies have been developed, including the Super-Twisting Algorithm (STA), Sliding Mode Control (SMC), Backstepping control, and Fractional-Order PID control. These approaches have demonstrated notable advantages in improving system robustness, disturbance rejection, and fault ride-through capability [37–39].

The coordinated scheduling of wind power and energy storage systems has emerged as a key research direction in recent years, as it offers an effective pathway to enhance the efficient utilization of wind energy. To address the impact of wind speed fluctuations on power output, researchers have proposed energy allocation strategies based on Integral Sliding Mode Control (ISMC). These strategies enable real-time adjustment of charging and discharging behaviors in energy storage systems, thereby smoothing wind power output and ensuring the safe and stable operation of batteries [40–42]. In addition, in the field of equipment condition monitoring and fault diagnosis, scholars have developed fault identification methods for asynchronous motor bearings based on the Hilbert transform. These methods are often combined with Artificial Neural Networks (ANNs) and Fuzzy Logic Control (FLC) to enhance diagnostic accuracy and robustness, contributing significantly to the construction of proactive operation and maintenance systems for IES [43].

At the system integration level, substantial progress has also been made in the exploration of multi-energy complementary hybrid systems. Typical configurations include deeply integrated wind–solar–hydrogen–storage/fuel cell systems [44], which leverage the coordinated operation and complementary characteristics of multiple energy carriers to enhance system stability and overall energy efficiency. In response to the growing trend of transportation electrification, researchers have also proposed photovoltaic-based electric vehicle charging optimization models that account for both grid-connected and islanded operation modes, aiming to achieve cost-optimal and secure operation simultaneously [45]. Regarding energy storage health management, recent studies have introduced advanced algorithms—such as the Bald Eagle Search (BES) and Gradient-Based Optimization (GBO)—for accurate estimation of battery capacity and State of Health (SOH), providing effective tools to improve lifetime prediction accuracy and enable intelligent maintenance scheduling [46,47].

In summary, while prior research has made significant progress in the technical modeling and control of IES, a critical gap remains in aligning IES operation with evolving policy mechanisms such as CET and GCT. Existing studies often treat policy variables as exogenous, overlook dynamic market volatility, and fail to capture the full system-level benefits of IES under multi-dimensional performance goals. Moreover, current CET and GCT frameworks are not well-suited to the multi-energy coupling nature of IES, leading to challenges in emission accounting, value recognition, and coordinated decision-making. Addressing these issues requires an integrated modeling approach that embeds institutional mechanisms and supports resilient, policy-compliant IES operation.

## 3 CET and GCT mechanisms

### 3.1 Tiered CET mechanism

The CET mechanism refers to a trading system that achieves carbon emission control through the establishment of a legitimate CET accreditation mechanism and permits its buying and selling [48–50]. Enterprises adjust their production plans based on carbon quotas set by the government or regulatory bodies. If carbon emissions during the operational process surpass the allocated quotas, additional credits must be procured from the CET market; inversely [51–53], if emissions fall below the quota, surplus credits can be sold for profit [54]. Presently, carbon quotas are predominantly distributed free of charge within the domestic framework. This paper employs the baseline method and the pre-allocation approach to establish non-compensatory carbon quotas within the IES. The primary sources of carbon emissions in the IES, such as gas turbines and gas boilers, have their free carbon quotas calculated using Equation (1) [55].


$$\left\{ \begin{array}{c} @l@C_{L}=C_{q}+C_{h}\\ C_{q}=\overset{t}{\underset{g}{P}}B_{g}F_{e}F_{r}F_{f}\\ C_{h}=\overset{t}{\underset{b}{Q}}B_{h} \end{array}\right.$$ (1)

In the equation: $C_{L}$ represents the free carbon quota; $C_{q}$ represents the carbon quota for gas turbines; $C_{h}$ represents the carbon quota for gas boilers;$\overset{t}{\underset{g}{P}}$ represents the power output of the gas turbine at time t;$B_{g}$ is the carbon emission baseline for gas turbines, expressed in units of tons of $CO_{\text{2}}$t/(MW·h); $F_{e}$ is the cooling mode correction factor for gas turbines, with a value of 1 for water cooling and 1.05 for air cooling; $F_{t}$ is the heating correction factor for gas turbines, which can be calculated based on the heat-to-power ratio $\alpha_{GT}$; $F_{f}$ is the load (output) correction factor for gas turbines; $\overset{t}{\underset{b}{Q}}$ represents the power output of the gas boiler at time t; $B_{h}$ is the carbon emission baseline for gas boilers, expressed in units of tons of $CO_{\text{2}}$ (t/GJ).

The actual carbon emissions $C_{p}$ of the IES are determined by the output of the gas turbines and gas boilers, as shown in Equation (2) [56]:


$$C_{p}=\sum_{t=1}^{h}\left[a_{\text{1}}+b_{\text{1}}\overset{t}{\underset{g}{P}}+c_{\text{1}}{\left(\overset{t}{\underset{g}{P}}\right)}^{\text{2}}\right]+\sum_{t=1}^{h}\left[a_{\text{2}}+b_{\text{2}}\overset{t}{\underset{b}{Q}}+c_{\text{2}}{\left(\overset{t}{\underset{b}{Q}}\right)}^{\text{2}}\right]$$ (2)

In the equation: $a_{\text{1}}$, $b_{\text{1}}$ and $c_{\text{1}}$ represent the calculation coefficients for carbon emissions from gas turbines; $a_{\text{2}}$, $b_{\text{2}}$ and $c_{\text{2}}$ represent the calculation coefficients for carbon emissions from gas boilers;h represents the total number of scheduling intervals, expressed as h=24.

The tiered CET cost calculation model is formulated as follows [57]:


$$C_{CO_{\text{2}}}=\left\{ \begin{array}{c} @l@\lambda \left(C_{p}-C_{L}\right),C_{p}\leq C_{L}+d\\ \lambda \left(1+\lambda \right)\left(C_{p}-C_{L}-d\right)+\lambda d,C_{L}+d<C_{p}\leq C_{L}+2d\\ \lambda \left(1+2\sigma \right)\left(C_{p}-C_{L}-2d\right)+\lambda d\left(2+\sigma \right),C_{L}+2d<C_{p}\leq C_{L}+3d\\ \lambda \left(1+3\sigma \right)\left(C_{p}-C_{L}-3d\right)+\lambda d\left(3+\sigma \right),C_{L}+3d<C_{p}\leq C_{L}+4d\\ \lambda \left(1+4\sigma \right)\left(C_{p}-C_{L}-4d\right)+\lambda d\left(4+\sigma \right),C_{p}>C_{L}+4d \end{array}\right.$$ (3)

In the formula: $C_{CO_{\text{2}}}$ represents the CET cost for the IES, λ is the market price of CET, d is the length of the carbon emissions interval, and σ is the incremental increase in CET price per tier. As the tier increases, the price of CET grows by σλ. If$C_{P}<C_{L}$, $C_{CO_{\text{2}}}$ is negative, it indicates that the CET results in a financial gain.

### 3.2 GCT mechanism

GCT involves issuing certificates to renewable energy producers, certifying that a portion of their electricity generation originates from renewable sources [52]. This mechanism acts as a supportive measure to facilitate the effective implementation of RPS [58]. The primary objective of deploying both RPS and GCT mechanisms is to shift the subsidization of renewable energy from direct governmental subsidies to market-driven incentives, fostering a gradual transition towards sustainable energy practices [59]. When the quantity of GCT obtained by the IES exceeds the minimum renewable consumption obligation, the surplus certificates can be sold for revenue [60]. Conversely, if the IES falls short of this minimum, additional certificates must be purchased from the green certificate market [61]. Consequently, the calculation model for GCT is as follows:


$$\begin{array}{c} \begin{array}{c} C_{gre}=\left\{ \begin{array}{cc} @cc@\frac{P_{w}-P_{res}}{1000}\overset{b}{\underset{gre}{c}}-C_{f}\left(P_{res}-P_{w}\right) & P_{w}<P_{res}\\ \frac{P_{w}-P_{res}}{1000}\overset{s}{\underset{gre}{c}} & P_{w}\geq P_{res} \end{array}\right. \end{array} \end{array}$$ (4)

In the formula, $\overset{b}{\underset{gre}{c}}$,$\overset{s}{\underset{gre}{c}}$ respectively represent the prices for buying and selling green certificates; $P_{w}$ is the actual consumption of renewable energy; $P_{res}$ is the daily quota of renewable energy consumption, and $C_{f}$ is the penalty coefficient.

### 3.3 Joint trading of CET and GCT

**Fig 2:**
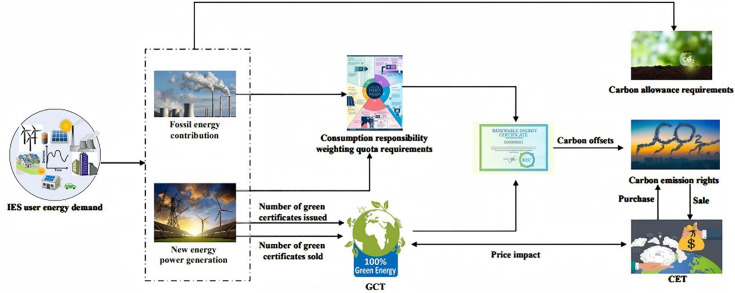
Schematic diagram of joint trading between carbon and green certificate markets.

Fig 2 illustrates the coordinated trading architecture that links the CET market with the GCT market and shows how a single megawatt-hour of renewable generation produces two distinct but traceable credits.

After the monetary settlement of power flows, the GCT platform validates each unit of renewable output and issues (i) a green certificate GC that proves compliance with the renewable-portfolio mandate and (ii) a quantified amount of verified CO₂ abatement benchmarked against the regional grid-emissions factor. The GC enters the certificate market where electricity retailers and large consumers purchase it to meet statutory renewable-consumption quotas. The matched CO₂ abatement value is uploaded to the CET registry and can be surrendered by emitting entities to offset part of their regulated carbon liability.

#### (1) Price signals travel in both directions, creating a feedback loop between the two markets.

When the GC price rises, renewable producers obtain higher revenue and expand output. The additional generation increases the volume of certified abatement, which in turn enlarges the supply of CET allowances and eases upward pressure on the carbon-permit price. Lower permit prices reduce compliance costs for fossil generators and other emitters, helping to stabilize system-wide electricity costs. In the opposite direction, a higher CET price strengthens demand for low-carbon certificates by making fossil generation more expensive, which improves the liquidity and depth of the GC market.

#### (2) The architecture incorporates a reconciliation layer that prevents double counting and limits arbitrage.

Every certificate issued by the GCT platform is cross-checked against the CET ledger before an abatement credit is released. Shared data interfaces, real-time monitoring, and coordinated enforcement ensure that the CET cap trajectory remains consistent with the renewable-deployment targets embedded in the RPS. Where price signals and compliance options are properly aligned, the two schemes reinforce one another. If either market is poorly enforced or subject to unexpected shocks, participants can shift toward the cheaper instrument, which weakens overall climate performance. Proper governance therefore hinges on transparent data exchange, harmonized penalty rules, and periodic recalibration of the cap and quota parameters.

## 4 Wind-solar-gas-storage IES framework

### 4.1 The framework of IES

**Fig 3:**
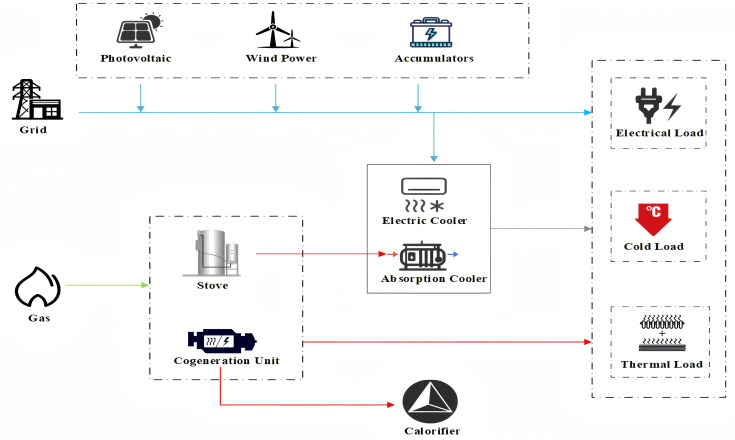
The structure of the IES.

The structure of the IES investigated in this study is illustrated in Fig 3, which includes energy supply equipment, energy production equipment, and energy storage devices. The energy demand encompasses electrical, heating, and cooling loads (The pseudocode for the research framework can be found in Appendix Table B in S3 Appendix).

### 4.2 Power models of key equipment in user energy systems

In user energy systems, the physical structures and operational characteristics of various units and devices vary significantly. Considering that this study primarily focuses on the planning and operational optimization of user energy systems, transient processes of various units and devices during operation are not considered. Therefore, this research disregards their transient response processes. Based on the power characteristics and operating conditions of various units and devices, a general power model is established (A summary of all variables is provided in Appendix **Table A** in S3 Appendix).

#### 4.2.1 Renewable energy generation systems.

(1) Power model of wind turbines.

Wind power generation harnesses renewable wind energy to produce electricity. The output power of a wind turbine is closely related to the wind speed. The relationship between the output electrical power and the wind speed can be represented by a piecewise function [62]:


$$\begin{array}{c} P_{wt}=\left\{ \begin{array}{c} @c@\begin{array}{ccc} 0 & & 0\leq v\leq v_{in} \end{array}\\ \begin{array}{ccc} W_{wt}\frac{v-v_{in}}{v_{r}-v_{in}} & & v_{in}<v\leq v_{r} \end{array}\\ \begin{array}{ccc} W_{wt} & & v_{r}<v\leq v_{out} \end{array}\\ \begin{array}{ccc} 0 & & v_{out}\leq v \end{array} \end{array}\right. \end{array}$$ (5)

In the formula, $P_{wt}$ and $W_{wt}$ respectively represent the actual power and rated power of the wind turbine;v,$v_{in}$,$v_{r}$ and $v_{out}$correspondingly represent the actual wind speed, cut-in wind speed, rated wind speed, and cut-out wind speed.

(2) Power model of photovoltaic cells.

Photovoltaic power generation involves converting renewable solar energy into electrical energy. The amount of electricity generated is primarily dependent on temperature and solar irradiance. The relationship between the output electrical power and both temperature and solar irradiance can be represented by a piecewise function [63]:


$$P_{pv}=P_{stc}\frac{G_{c}}{G_{stc}}\left[1+\gamma \left(T_{c}-T_{stc}\right)\right]$$ (6)

In the formula, $P_{pv}$ and $P_{stc}$ respectively represent the actual power and rated power of the photovoltaic (PV) modules;$G_{c}$and $G_{stc}$ respectively denote the solar irradiance during project operation and under standard conditions;γ is the power temperature coefficient; $T_{c}$ and $T_{stc}$ respectively represent the actual temperature and the rated temperature.

#### 4.2.2 CCHP systems.

CCHP systems integrate refrigeration, heating, and power generation into a single system that can simultaneously produce electricity, thermal energy, and cooling energy. These systems not only facilitate the cascading use of energy but also reduce the emissions of pollution gases such as CO_2_ and PM_2.5_, thereby contributing to a low-carbon economy. The typical architecture of a CCHP system includes a Power Generation Unit (PGU), heating devices, and cooling devices. CCHP systems commonly utilize natural gas or biogas as fuel and are located close to the user side, forming a major subsystem of user energy systems. By coordinating with renewable energy systems and storage systems, CCHP systems meet the user-end demands for electric, cooling, and heating loads.

The PGU is the core equipment of the CCHP system, capable of simultaneously generating electricity and thermal energy. PGUs used in CCHP systems include Stirling engines, gas turbines, micro gas turbines, internal combustion engines, and fuel cells. Among these, micro gas turbines, typically ranging from several tens to hundreds of kW in power and known for their compact size, high power output, and mature technology, are selected for use in this study as the PGU within the user energy system. For PGU units, this paper sets a minimum load rate of 0.25, and the power generation efficiency of PGU units varies with the load rate [64].


$$P_{PGU}=Q_{PGU}\eta_{e}$$ (7)

$$\eta_{e}=-0.85{\left(\frac{P_{PGU}}{P_{PGU,max}}\right)}^{\text{2}}+0.15exp\left(-5\frac{P_{PGU}}{P_{PGU,max}}\right)+0.05+0.02sin\left(t\right)$$ (8)

$$0.25U_{PGU}\left(t\right)P_{PGU,max}\leq P_{PGU}\left(t\right)\leq U_{PGU}\left(t\right)P_{PGU,max}$$ (9)

In the formula:$Q_{PGU}$ represents the heat input to the PGU; $\eta_{e}$ denotes the power generation efficiency of the PGU; $P_{PGU,max}$is the maximum output power of the PGU; $U_{PGU}$ indicates the operational status of the PGU at time *t*, where 0 represents shutdown, and 1 represents operation.

#### 4.2.3 Energy storage output model.

Energy storage technology plays a crucial role in microgrid systems, but selecting the appropriate storage technology depends on the specific needs of the project. Lithium iron phosphate (LiFePO4) batteries, due to their inherent characteristics, form a central component of the entire photovoltaic and wind power storage microgrid system and are currently among the most widely used in the energy storage field.

The energy level of a storage system is dynamic and generally indicated by the State of Charge (SOC). The SOC of a lithium iron phosphate battery during charging and discharging cycles is influenced by the residual energy from the preceding moment and its current charge or discharge power. During the charging phase, the SOC at any given time *t* can be expressed by the following equation (10) [65]:


$$SOC\left(t\right)=SOC\left(t_{\text{0}}\right)+\overset{ch}{\underset{BS}{\eta }}\frac{\overset{ch}{\underset{BS}{P}}\left(t_{\text{0}}\right)}{C_{bat}}\Delta t$$ (10)

When a lithium iron phosphate battery discharges, the SOC at time *t* can be represented by the following equation (11):


$$SOC\left(t\right)=SOC\left(t_{\text{0}}\right)+\frac{\overset{dis}{\underset{BS}{P}}\left(t_{\text{0}}\right)}{\overset{dis}{\underset{BS}{\eta }}C_{bat}}\Delta t$$ (11)

In the formula:$\overset{ch}{\underset{BS}{\eta }}$ and $\overset{dis}{\underset{BS}{\eta }}$ respectively represent the charging and discharging efficiencies of the lithium iron phosphate battery; $\overset{ch}{\underset{BS}{P}}\left(t_{\text{0}}\right)$ and $\overset{dis}{\underset{BS}{P}}\left(t_{\text{0}}\right)$ are the charging and discharging power of the lithium iron phosphate battery at time $t_{\text{0}}$, expressed in kW; $C_{bat}$ is the storage capacity of the lithium iron phosphate battery, expressed in kWh.

## 5 Economic operation optimization model of IES considering CET and GCT

*The economic operation problem in§5 is posed from the perspective of a price taking IES operator. Accordingly, the carbon allowance price* $P^{CE\text{T}}$ *and green certificate price* $P^{GC}$ *are treated as exogenous inputs to the optimization and in the subsequent sensitivity analysis. Section* *3.3* *provides a market level conceptual link between the CET and GC markets (e.g., how a higher GC price can expand renewable output and ease CET prices), but this feedback is not endogenized in the MILP; instead, we probe operator responses across a grid of* $\left(P^{CET},P^{GC}\right)$ *scenarios to reflect plausible market conditions.*

### 5.1 Objective function

This study aims to minimize the total costs of the IES, encompassing the costs associated with purchasing electricity, purchasing gas, engaging in carbon and green certificate trading, and the operation and maintenance of other equipment within the system. We clarify the underlying reasoning behind unifying various cost elements into a single total cost metric in our optimization framework.

In this study, we adopt an integrative approach that aggregates grid electricity costs, natural gas expenses, carbon quota fees, and green certificate transactions into a single optimization objective. This unified perspective allows us to capture the net economic impact on the IES operator, reflecting the true cost trade-offs among diverse energy resources and policy instruments.

The modeling framework centers on an IES operator (or aggregator) responsible for coordinating energy generation, storage, and demand within the system boundary. By assuming a single decision-maker, we can precisely track how costs and revenues flow within the IES, thereby simplifying the analysis and enhancing interpretability of the optimization outcomes.

The IES operator may be subject to multiple market mechanisms simultaneously, including the purchase (or sale) of electricity from the grid, procurement (or trade) of carbon quotas, and acquisition (or trading) of green certificates. Treating these components within one objective function ensures that the operator’s scheduling and investment decisions holistically reflect all relevant market signals, facilitating more accurate cost optimization and compliance with environmental constraints.

External generation costs—such as those incurred by conventional power stations—are already embedded in the retail electricity tariff, which is the rate at which the IES operator buys power from the grid. Consequently, the model assumes that purchasing electricity at the specified tariff implicitly covers upstream production and transmission expenses. This approach prevents double-counting and preserves a streamlined focus on the direct costs actually borne by the IES operator.

The objective function to achieve this goal can be expressed as follows:


$$min\;C=C_{e}+C_{gas}+C_{es}+C_{hs}+C_{CO_{\text{2}}}-C_{gre}$$ (12)

(1) Cost of purchasing electricity:

$$C_{e}=\sum_{t=1}^{h}\Delta tP_{grid}\left(t\right)C_{grid}\left(t\right)$$ (13)

In the formula:$P_{grid}\left(t\right)$ represents the power purchased from the grid during time period *t*; $c_{grid}\left(t\right)$ is the unit cost of elec*t*ricity at time *t*; and Δt denotes the duration of the adjustment period [66].

(2) Cost of purchasing gas:

$$C_{gas}=c_{gas}\sum_{t=1}^{h}\left(\frac{P_{PGU}\left(t\right)}{\eta_{e}}\right)\Delta t$$ (14)

In the formula:$c_{gas}$ represents the unit price of natural gas per unit of calorific value; Δt denotes the duration of the adjustment period.

(3) Battery operating costs:

Assuming that the cost of a single charge and discharge cycle is the same for lithium iron phosphate batteries, if the purchase cost is $C_{purchase}$, and the battery can be used for M cycles without damage, the cost $C_{r}$ of per complete charge and discharge cycle can be calculated as follows:


$$C_{r}=\frac{C_{purchase}}{M}$$ (15)

The operating and maintenance cost for lithium iron phosphate batteries can be calculated:


$$C_{es}=\sum_{t=1}^{h}C_{r}\frac{\overset{ch}{\underset{BS}{P}}\left(t\right)/\overset{dis}{\underset{BS}{P}}\left(t\right)}{C_{bat}}$$ (16)

In the formula, $\overset{ch}{\underset{BS}{P}}\left(t\right)$ and $\overset{dis}{\underset{BS}{P}}\left(t\right)$ respectively represent the charging and discharging power of the lithium iron phosphate battery at timet [67].

(4) Operating and maintenance costs of thermal storage equipment

$$C_{hs}=\sum_{i=1}^{h}\overset{t}{\underset{hs}{c}}\left(\overset{t}{\underset{hs,c}{Q}}+\overset{t}{\underset{hs,d}{Q}}\right)\Delta t$$ (17)

In the formula, $\overset{t}{\underset{hs}{c}}$ represents the operating and maintenance cost of the thermal storage equipment at timet;$\overset{t}{\underset{hs,c}{Q}}$ and $\overset{t}{\underset{hs,d}{Q}}$ respectively denote the charging and discharging thermal power of the thermal storage equipment at timet.

(5) The costs associated with CET within the IES are calculated as detailed in Equation (3).
(6) The costs related to GCT are specified in Equation (4).

### 5.2 Constraints

#### 5.2.1 Equipment constraints.

During the operation of the IES, it is essential to consider the energy conversion relationships and capacity constraints of each piece of equipment. These constraints ensure that the equipment operates within its designed parameters to prevent overloads and maintain efficiency. The energy conversion constraints for each device have already been explained in Section 3.2.

#### 5.2.2 Power balance constraints.

The constraints for balancing electric, thermal, and cooling loads are as follows:


$$P_{grid}\left(t\right)+P_{pv}\left(t\right)+P_{wt}\left(t\right)+P_{PGU}\left(t\right)+\overset{ch}{\underset{BS}{\eta }}\overset{ch}{\underset{BS}{P}}\left(t\right)-\overset{dis}{\underset{BS}{P}}\left(t\right)/\overset{dis}{\underset{BS}{\eta }}-P_{EC}\left(t\right)=P_{e,load}\left(t\right)$$ (18)

$$P_{GB}\left(t\right)+Q_{PGU}\left(t\right)-P_{AC}\left(t\right)\geq Q_{h,load}\left(t\right)$$ (19)

$$\overset{*}{\underset{AC}{P}}\left(t\right)+\overset{*}{\underset{EC}{P}}\left(t\right)\geq Q_{c,load}\left(t\right)$$ (20)

In the formula: $P_{AC}$ represents the input power to the absorption chiller, $P_{GB}$ is the output power of the boiler,$\overset{*}{\underset{AC}{P}}$ and $\overset{*}{\underset{EC}{P}}$respectively are the output powers of the absorption chiller and the electric chiller; $Q_{h,load}$ and $Q_{c,load}$ are the thermal and cooling loads of the IES.

#### 5.2.3 Caps on the selling price of green certificates.

The minimum selling price in the green certificate market should be set at a level equivalent to the present value of the funds supplemented by the subsidy for renewable energy electricity prices. Conversely, the maximum selling price should not exceed the difference between the on-grid electricity price of renewable energy for the corresponding volume and the benchmark electricity price of a gas turbine. Therefore, the pricing structure in the green certificate market is bounded by the following minimum and maximum caps:


$$\overset{min}{\underset{gre}{C}}=\frac{1000\left(s_{i}-c\right)}{{\left(1+r_{i}\right)}^{h_{i}+d_{i}}}$$ (21)

$$\overset{max}{\underset{gre}{c}}=1000\left(s_{i}-c\right)$$ (22)

In the formula, $\overset{min}{\underset{gre}{c}}$ and $\overset{max}{\underset{gre}{c}}$ r epresent the minimum and maximum price limits in the market, respectively. $s_{i}$ is the on-grid electricity price fori certificates under the GCT scheme, c is the benchmark electricity price of a gas turbine. $r_{i}$ is the discount rate for thei type of green energy, $h_{i}$ is the subsidy settlement cycle for thei type of green energy electricity price, and $d_{i}$ is the deferred payment period for the subsidy amount for thei type of green energy [68].

#### 5.2.4 Green certificate quota constraints.


$$\sum_{i=1}^{v}G\alpha_{i}P_{i}-G_{gre}=G\sum_{i=1}^{v}\pi_{i}P_{io}$$ (23)

In the equation, G represents the quantification coefficient, which is the number of green certificates that can be obtained per unit of green electricity produced. $\alpha_{i}$ is the proportion of renewable energy generation by the GCT party within a given time frame, $P_{i}$ denotes the actual electricity generation by the GCT party, $G_{gre}$ is the number of green certificates, $\pi_{i}$ is the renewable energy quota coefficient for the GCT party within the specified period, and $P_{io}$ is the initial allocated electricity volume for the GCT party [69].

## 6 Simulate models through practical applications

In order to rigorously validate the effectiveness and real-world applicability of the proposed optimization model, this study employs a representative case drawn from a Chinese IES. China is intentionally selected as the focal research context for three main reasons, reflecting both policy representativeness and data richness.

First, China’s energy market is currently undergoing a profound transformation characterized by the rapid evolution of both the CET and GCT mechanisms. These two market instruments have reached nationwide coverage, encompassing multiple industries and provinces, and are subject to continual institutional refinement. The coexistence of both schemes within a single policy environment creates a unique natural laboratory for examining the synergistic interactions between emission pricing and renewable-energy incentives-conditions that are rarely observed concurrently in other jurisdictions.

Second, the gradual liberalization of China’s power market—together with the growing maturity of real-time electricity pricing, ancillary service trading, and coordinated CET-GCT policies—provides abundant, high-resolution operational data. This policy and data landscape enables the construction of stochastic simulation scenarios that closely mirror actual market operations. In particular, historical datasets of wind speed, solar irradiance, and electricity prices were used to generate Monte Carlo samples capturing their inherent volatility. These samples were subsequently reduced via a k-medoids clustering algorithm to form representative scenarios for the two-stage stochastic–robust optimization model. Consequently, the model explicitly accounts for renewable intermittency, market price uncertainty, and their joint impact on IES operation through expected-cost and Conditional Value-at-Risk (CVaR) evaluation.

Third, China’s large-scale deployment of IES projects—ranging from urban industrial parks to distributed multi-energy microgrids—offers a highly diverse set of operating environments. Variations in regional climate, energy mix, and load structure generate a broad spectrum of electricity, heating, and cooling demands as well as equipment configurations. This diversity ensures that the model’s outcomes capture not only local dynamics but also system behaviors representative of other regions undertaking similar multi-energy integration initiatives.

Therefore, focusing on a Chinese case does not limit the generality of the research; rather, it aligns the analysis with a context that is both data-rich and globally relevant. China’s hybrid of mature regulatory design, policy experimentation, and heterogeneous operating conditions provides a valuable proving ground for validating the proposed stochastic–robust optimization framework. The methodological structure and parameterization are fully transferable to other jurisdictions pursuing comparable low-carbon transitions and market-based policy reforms.

**Fig 4:**
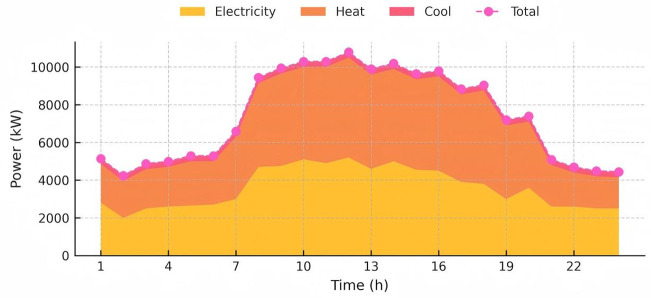
The heat, cool, gas and electricity load demand of the IES.

**Fig 5:**
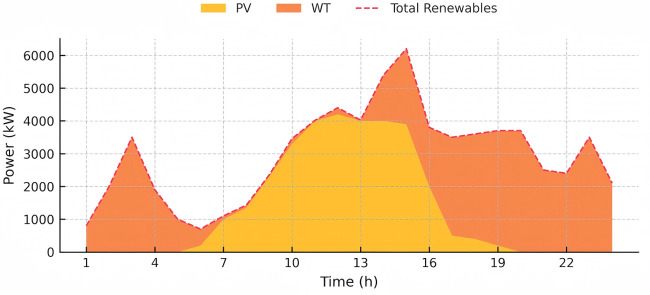
The PV and WT output prediction curve of the IES.

**Fig 6:**
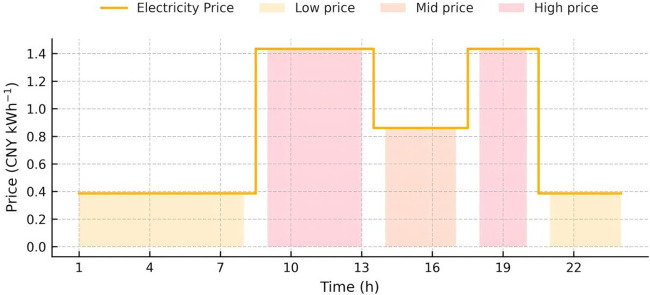
Prices of the electricity.

The capacity and parameters of the equipment utilized are detailed in Table 1. To assess the long-term benefits of the proposed model, day-ahead scheduling was set for 24 periods, with each period lasting one hour. During the solution process, the system’s carbon emissions were linearized in a piecewise manner, transforming the scheduling model for each interval into a Mixed Integer Linear Programming (MILP) problem. The simulations were performed using YALMIP and CPLEX. Considering that the GCT mechanism is still evolving, the price of green certificates was set at 100 CNY per certificate, and the CET price was set at 0.15 CNY/kg. Data on the system’s cooling, heating, electrical loads, photovoltaic outputs, wind turbine data, and real-time electricity prices are provided in Fig 4, Fig 5 and Fig 6, respectively, with natural gas priced at 0.35 CNY/kWh.

**Table 1:** Main parameters of the equipment.

Equipment	Parameters	Value
**Combined heat and power**	$P_{grid}$ $P_{PGU}$ $Q_{PGU}$	4500KW 8000KW 5500KW
**Gas boiler**	$P_{GB}$	6500KW
**Battery storage**	$P_{BS}$ $\overset{ch}{\underset{BS}{\eta }}/\overset{dis}{\underset{BS}{\eta }}$	2500KW 0.89
**Wind turbine**	$P_{wt}$	6000KW
**Photovoltaic (PV) panels**	$P_{pv}$	6700KW
**Electric chiller**	$P_{EC}$	1800KW
**Absorption chiller**	$P_{AC}$	1800KW
**Thermal storage tank**	$Q_{hs}$	50000KW

To ensure practical applicability and computational tractability, the model was implemented on a standard workstation (Intel Core i7-12700, 32 GB RAM, Windows 11, MATLAB 2022b). The complete MILP formulation typically consists of approximately 8,000–15,000 decision variables and 5,000–10,000 constraints, depending on the number of Monte Carlo scenarios retained after reduction. The average solution time for a 24-hour horizon with 50 scenarios is approximately 3.2 minutes, with a worst-case solution time (100 scenarios, full resolution) of under 7 minutes. These runtimes demonstrate that the framework is efficient and well-suited for day-ahead or intra-day operational planning contexts.

To manage the complexity induced by uncertainty and market interactions, several computational techniques were employed: We applied a k-medoids clustering algorithm to condense the initial Monte Carlo samples into a manageable set of representative CET and GCT price trajectories while preserving the underlying distributional features. This significantly reduced the scenario tree size and improved convergence. The tiered carbon quota pricing mechanism and green certificate quota cost structures were modeled using constraints, which are natively supported and highly optimized in CPLEX. This approach allows nonlinear pricing functions to be embedded into MILP efficiently. The model architecture exploits weak temporal coupling between time intervals for most physical subsystems, enabling future application of rolling-horizon optimization or parallel computation methods. Constraint matrices were structured to maintain sparsity in both energy balance and emission quota constraints. This accelerates LP relaxations and enhances the branching performance of the MILP solver.

In terms of scalability, preliminary stress testing demonstrated that: Doubling the number of time periods (from 24 to 48) increases computation time by a factor of ~2.5, indicating polynomial time scaling rather than exponential. Increasing the number of scenarios from 50 to 100 increases computation time by ~1.9 × , demonstrating good linear scalability with respect to scenario size. The framework remains stable even under full-coupling assumptions (e.g., inter-temporal carbon credit rollover), although solution time increases accordingly.

### 6.1 Sensitivity analysis in different scenarios

To examine the impact of carbon trading and green certificate trading on the IES, this study delineates four distinct scenarios:

Scenario 1: The IES does not incorporate CET or GCT.
Scenario 2: The IES incorporates CET but does not consider GCT.
Scenario 3: The IES incorporates GCT but does not consider CET.
Scenario 4: The IES incorporates both CET and GCT

To make the experimental design transparent, we now specify the two layers of scenarios used in the paper (Shown in Table 2 and Table 3):

**Table 2:** Policy-mix layer.

Scenario ID	Market instruments active	Reference prices*	Purpose
S1	None	—	Baseline for cost and emission comparisons
S2	CET only	Carbon = 0.15CNYkg ⁻ ¹	Isolate impact of carbon pricing
S3	GCT only	GC = 100CNYcertificate ⁻ ¹	Isolate impact of green certificate incentives
S4	CET + GCT	Carbon = 0.15CNYkg ⁻ ¹; GC = 100CNYcertificate ⁻ ¹	Assess policy synergy

* Reference prices mirror mid-2023 averages in China’s pilot carbon market and regional GC spot market

**Table 3:** Price-sensitivity layer.

Sweep variable	Range tested	Step	Result subsection
**Carbon price (CNYkg ⁻ ¹)**	0 → 0.35	0.05	§5.2&§5.3
**GC price (CNYcertificate ⁻ ¹)**	30 → 100	10	§5.4

**Fig 7:**
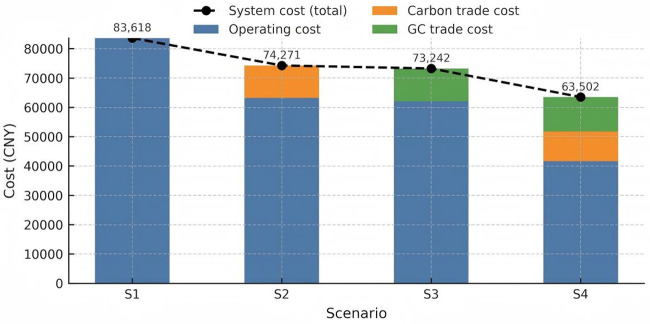
The cost of different scenarios.

**Fig 8:**
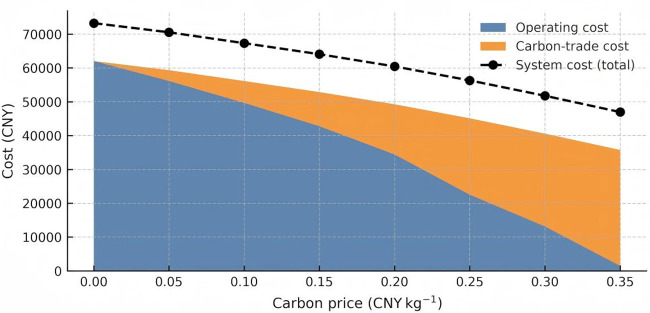
Composition of system cost as a function of carbon price.

**Fig 9:**
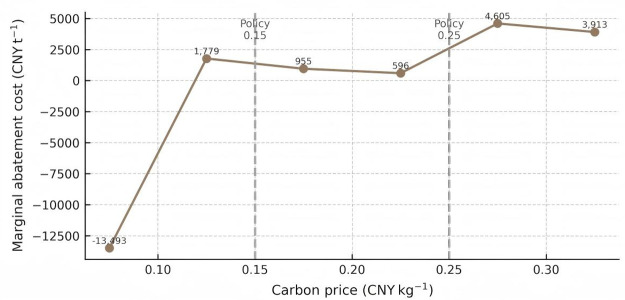
Marginal abatement cost curve with two illustrative policy thresholds.

**Fig 10:**
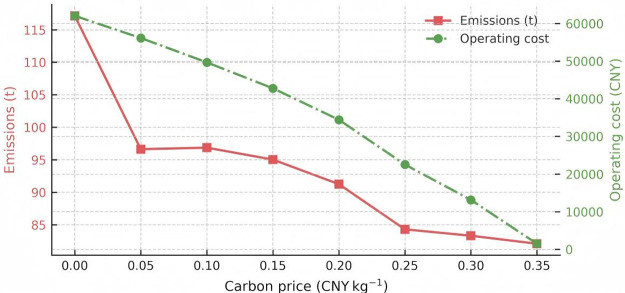
Trade off between emissions (left axis) and operating cost (right axis).

**Fig 11:**
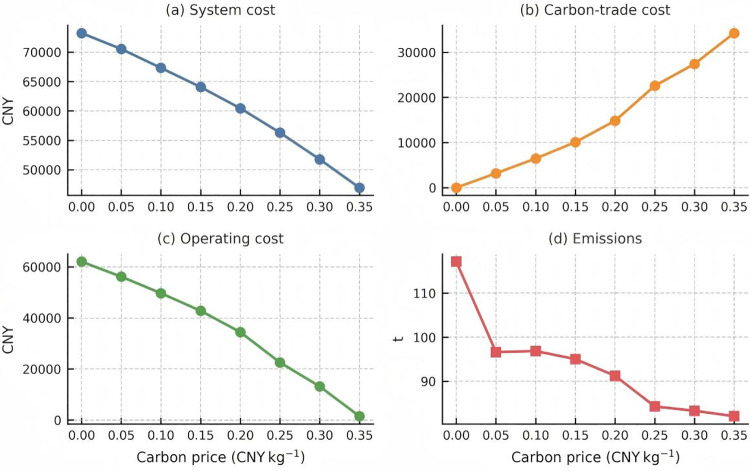
Carbon price response of key system metrics. Combined Fig 11: (a) System cost, (b) Carbon trade cost, (c) Operating cost, (d) Emissions. Panel (a) confirms the near linear decline in total system cost, which drops by 36 percent as the carbon price increases from 0 to 0.35 CNY kg ⁻ ¹. Panel (b) reveals the nonlinear escalation of carbon trade expenditure. The trade payment remains modest below 0.15 CNY kg ⁻ ¹ but more than doubles once the price surpasses 0.25 CNY kg ⁻ ¹. Panel (c) illustrates the rapid erosion of operating outlays, already halved at 0.15 CNY kg ⁻ ¹ and virtually eliminated above 0.30 CNY kg ⁻ ¹. Panel (d) shows direct CO₂ emissions falling by roughly 30 percent, which mirrors the cost response but with a smaller elasticity.

**Fig 12:**
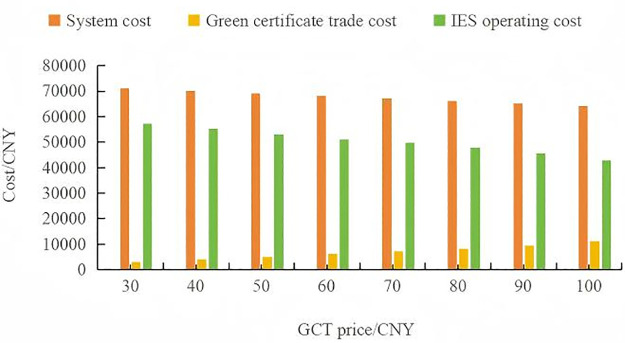
Impact of green certificate trading price changes on system costs. Panel (a) puts operating and certificate costs on separate axes and highlights their point of convergence. Operating cost dominates trading cost at all tested prices; however, the gap narrows from 47 kCNY at 30 CNY to just 32 kCNY at 100 CNY. Extrapolation suggests the two curves would intersect at approximately 140 CNY per certificate, after which the certificate expense becomes the larger cost driver. Panel (b) converts the cost curve into marginal savings: every ten‑CNY increment saves about 1 014 CNY up to 60 CNY, after which the incremental benefit plateaus at roughly 800 CNY. The flat slope beyond 70 CNY marks the onset of diminishing returns. Panel (c) reports a pay‑back ratio, defined as the operating‑cost saving divided by the extra trading expense for each price step. The ratio is roughly two in the 30–50 CNY corridor, indicating that every additional CNY spent on certificates releases two CNY of OPEX. The ratio drops to 1.2 for the 60 → 70 CNY step and falls below unity above 80 CNY. Once the ratio dips below one, further price increases merely reshuffle cost categories instead of reducing the overall burden.

**Fig 13:**
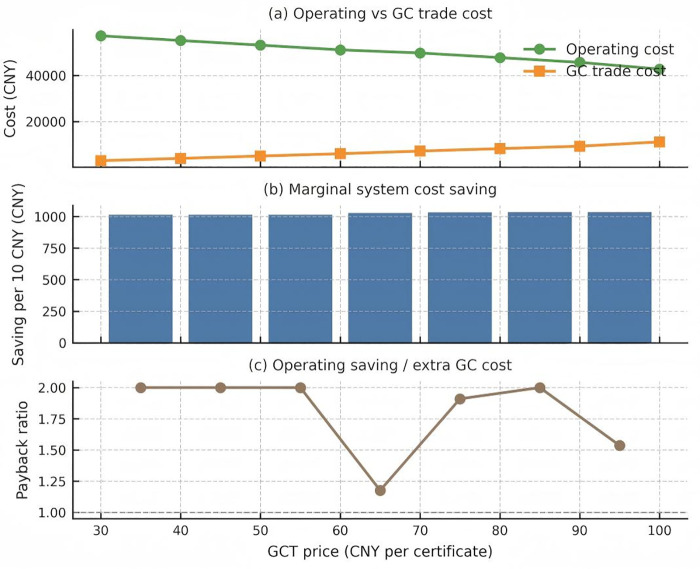
Impact detailed GCT metrics.

Layer 1 contrasts four static policy mixes (S1-S4) under the reference prices, generating the cost breakdown in Table 4 and Fig 7. Layer 2 performs one‑factor sweeps around S2 (carbon‑only) and S3 (GC‑only) to quantify how marginal price shocks affect total cost, operating cost, and emissions (Figs 8–13). Together, these layers show that the combined CET–GCT regime (S4) lowers operating cost by 26.2% versus single‑instrument cases and that the cost‑ and emission‑elasticities with respect to price are monotonic, confirming robustness across plausible market conditions.

**Table 4:** Cost summary for each scenario.

Scenario	System cost	Carbon trade cost	Green certificate trade cost	IES operating cost
**1**	83618.43	0	0	83618.43
**2**	74270.70	11108.01	0	63162.69
**3**	73242.15	0	11200.97	62041.18
**4**	63502.11	10084.88	11761.03	41656.20

These scenarios are designed to analyze how each trading mechanism individually, as well as their combination, affects the operational strategies, economic efficiency, and environmental outcomes of the IES. Compared with existing studies [70], this research adopts a more systematic and comprehensive approach to scenario design and price sensitivity analysis, enabling the identification of economically efficient zones and the quantitative demonstration of the synergistic effects of the dual mechanisms. Based on these findings, policymakers are advised to prioritize the coordinated implementation of CET and GCT mechanisms—for example, by establishing unified market pricing policies and designing policy portfolios that provide clear guidance on optimal pricing ranges—to achieve the dual objectives of cost reduction and emissions mitigation.

Referencing Table 4, the following interpretative narrative has been constructed:

Scenario 1 is posited as the foundational benchmark wherein costs from CET and GCT are absent, reflecting an operating cost commensurate with the system cost at 83,618.43 CNY. Transitioning to Scenario 2, it is observed that the integration of CET contributes to a marked reduction in the operating cost for the IES, registering at 63,162.69 CNY, which is a decrease of 20,455.74 CNY relative to Scenario 1. This is further augmented by carbon cost accruing to 11,108.01 CNY. It is imperative to note that in juxtaposition with Scenario 1, there is a discernible decrement in operating costs amounting to 10,389.79 CNY within Scenario 2.
Scenario 3 introduces an operational framework where GCT is accounted for in the absence of CET. The resulting system cost is marginally higher than that of Scenario 2, yet it boasts a lower operating cost of 62,041.18 CNY, supplemented by green certificate revenue of 11,200.97 CNY. When measured against Scenario 2, this scenario yields a modest operating cost saving of 1,121.51 CNY.
The fourth scenario encapsulates the concurrent application of both CET and GCT, culminating in the most pronounced curtailment of operating costs to 41,656.20 CNY. It is accompanied by a dual stream of cost from carbon, summing to 10,084.88 CNY, and from green certificates, contributing an additional 11,761.03 CNY. In a comparative light, Scenario 4 achieves a reduction in operating costs by 18,385.22 CNY (26.2%) relative to Scenario 3 and manifests an increase in green certificate revenue by 560.06 CNY in relation to Scenario 2.

Furthermore, the dualistic adoption of CET and GCT within Scenario 4 substantiates its superior economic performance. By synergistically harnessing both CET and GCT, the IES is optimized to reap enhanced financial returns, significantly attributed to the incentivization of renewable energy consumption that is eligible for both trading rewards. The scenarios incorporating CET, namely Scenarios 2 and 4, consistently illustrate a more pronounced decline in operating costs in contrast to Scenario 3, which exclusively factors in GCT. This observation suggests a more impactful role of carbon trading incentives in operational cost mitigation over sole reliance on green certificates. The additional cost generated from green certificates in Scenario 4 not only fortifies the financial rationale for augmenting renewable energy integration within the IES but also advances both economic and environmental objectives by facilitating a more effective reduction in greenhouse gas emissions. The specific stacked bar chart is shown in Fig 7. Compared with existing studies [71], this research leverages a real-world Chinese IES case and conducts detailed price sensitivity analysis to quantitatively demonstrate the dual benefits of coordinated policies—achieving over 26% cost reduction alongside effective emissions control—thereby offering stronger practical relevance. Policymakers should take into account the synergistic effects of the dual mechanisms when designing energy subsidies and incentive policies. For example, carbon pricing and green certificate pricing mechanisms should be jointly adjusted in accordance with regional energy structures and renewable energy penetration levels, in order to optimize system costs and maximize carbon reduction efficiency.

### 6.2 Impact of carbon trading price changes on system costs

Fig 8 decomposes the total system cost into its two movable components—operating cost and carbon‑trade cost—across the eight carbon‑price steps (0–0.35 CNY kg ⁻ ¹). A nearly linear downward trajectory is observed for the overall system cost (black dashed line), which falls by 36% from 73 242 CNY to 46 955 CNY. The decline is driven by a 97.6% reduction in operating expenditure, reflecting both improved plant‑dispatch efficiency and the progressive replacement of emission‑intensive gas‑turbine output. Conversely, the carbon‑trade outlay rises monotonically to 34 271 CNY, cancelling roughly one‑third of the operating‑cost savings at the highest price point. The stacked area vividly illustrates this “see‑saw mechanism”: each increment in carbon price simultaneously shifts value from OPEX to compliance cost, yet the net effect remains cost‑negative up to 0.35 CNY kg ⁻ ¹.

To probe the robustness of this trend, Fig 9 plots the marginal abatement cost (MAC) curve derived from successive cost and emission reductions. The MAC is negative (‑13 493 CNY t ⁻ ¹) for the first increment (0 → 0.05 CNY kg ⁻ ¹), signalling a “no‑regret” region where firms can simultaneously cut costs and emissions. Between 0.05 and 0.20 CNY kg ⁻ ¹ the MAC plateaus below 1 000 CNY t ⁻ ¹, a band often regarded as economically efficient in power‑sector modelling vertical grey bars mark policy‑relevant thresholds at 0.15 and 0.25 CNY kg ⁻ ¹. Beyond 0.25 CNY kg ⁻ ¹, the MAC rises sharply (>3 800 CNY t ⁻ ¹), implying that further emissions cuts become progressively more expensive and may warrant complementary subsidies or technology‑switch strategies.

Based on the above analysis, it is recommended that carbon pricing policies be set within the range of 0.05–0.20 CNY/kg to maximize the dual benefits of cost reduction and emissions mitigation, while avoiding the high-price region where the marginal abatement cost (MAC) increases rapidly. For scenarios where the carbon price exceeds 0.25 CNY/kg, complementary measures such as fiscal rebates, technology subsidies, or renewable energy investment incentives should be implemented to alleviate cost pressures and prevent compliance costs from negatively impacting system operations.

Our findings are highly consistent with carbon market analyses in China’s coal and power sectors. For instance, paper [72] used a differentiated modeling approach to show that pilot carbon trading schemes significantly increased the MAC for nuclear power plants (by approximately 120 CNY/ton), revealing the constraining effect of carbon markets on cost structures. Similarly, research by [73], based on industry- and province-level data, estimated that China’s short-term MAC averages around 20 USD/ton (approximately 140 CNY/ton). While this is lower than the MAC surge zone identified in our study (around 250 CNY/ton), both studies highlight the importance of maintaining a reasonable alignment between MAC and carbon pricing in order to ensure market efficiency and policy effectiveness [74,75].

### 6.3 Carbon price effects on emissions and operating costs

Fig 10 juxtaposes direct carbon emissions with operating cost on a dual‑axis layout. As the carbon price climbs to 0.35 CNY kg ⁻ ¹, emissions fall from 117 kt to 82 kt (‑29.9%), whereas operating cost plunges by 97%—a four‑fold larger elasticity (‑1.75 vs ‑0.40). The shaded zone between 0.10 and 0.20 CNY kg ⁻ ¹ underscores a sweet‑spot where incremental price signals continue to deliver meaningful abatement without imposing prohibitive compliance charges.

The four curves outline three policy relevant zones. First, a low price corridor at or below 0.05 CNY kg ⁻ ¹ represents a no regret region where marginal abatement costs are negative. Second, an economically efficient plateau between 0.05 and 0.20 CNY kg ⁻ ¹ balances falling operating costs against manageable trade payments. Third, a high price saturation region above 0.25 CNY kg ⁻ ¹ sees carbon trade costs accelerate and marginal abatement costs rise above 3 800 CNY per tone, which signals that complementary subsidies or revenue recycling may be required.

Based on the above analysis, policymakers are advised to implement complementary measures—such as fiscal subsidies, renewable energy investment incentives, or carbon price rebate mechanisms—when carbon prices exceed 0.25 CNY/kg, in order to prevent excessive compliance costs from undermining market efficiency. Our conclusions are consistent with, or provide support for, the carbon pricing logic found in existing literature. For example, although China’s current carbon price generally hovers around 50 CNY/ton (approximately 0.05 CNY/kg) [74], studies have shown that this range enables moderate emissions reductions while remaining economically feasible. In contrast, previous provincial-level MAC curve studies—such as those by Du Limin et al., which estimated China’s average MAC to be between 559 and 623 CNY/ton—suggest that achieving a 40–45% reduction in carbon intensity would result in a substantial increase in marginal costs, rising to around 0.6 CNY/kg [76]. Through simulation analysis, our study precisely identifies the “economically efficient zone” for carbon pricing and quantitatively captures the elasticity of both operating cost and emissions under a real-world Chinese IES case, thus offering more policy-relevant insights for effective carbon pricing design.

### 6.4 Impact of green certificate trading price changes on system costs

The Fig 12 illustrates three different cost metrics as functions of the GCT price in CNY. The GCT price, indicated on the horizontal axis, ranges from 30 to 100 CNY. The vertical axis represents various costs associated with the system, all presumably measured in CNY as well.

The system cost, shows a downward trend as the GCT Price increases. Beginning at 71,237.35 CNY when the GCT price is 30 CNY, it decreases to 64,062.15 CNY at a GCT price of 100 CNY. This suggests a negative correlation, indicating that higher GCT prices might incentivize lower system costs, perhaps due to more efficient energy production or consumption patterns.

The green certificate trade cost, also displays a linear increase with the rising GCT price. It starts at 3,041.80 CNY for a GCT price of 30 CNY and climbs to 11,200.98 CNY at a GCT price of 100 CNY. This positive correlation may imply that as the market price for green certificates rises, so does the cost of trading these certificates, potentially reflecting a higher demand for or the increasing value of green energy credits.

The IES operating cost, follows a similar descending trajectory as the system cost. The initial value is 57,180.19 CNY at a GCT price of 30 CNY, which falls to 42,776.30 CNY at a GCT price of 100 CNY. This decline indicates that higher GCT prices could be associated with reduced operating costs for an IES, possibly due to a shift towards more sustainable energy sources or more economical operating procedures.

In conclusion, the figure is expected to show that while the costs associated with green certificate trading rise with the GCT price, indicating possibly higher demand or value for sustainable practices, both the system and IES operating costs decrease. This could suggest that increasing GCT prices are effectively incentivizing reductions in overall and operational energy system costs, potentially by promoting investments in greener, more efficient technologies or practices (Fig 13).

Synthesizing all three diagnostics yields a three‑zone framework

Low‑price zone (30–50 CNY): high marginal savings and a pay‑back ratio near two make this range an economically attractive starting point for green‑certificate pricing.
Mid‑price zone (50–80 CNY): diminishing but positive returns justify moderate price ramps if the policy goal is to accelerate renewable uptake.
High‑price zone (>80 CNY): the pay‑back ratio falls below one; any further price hikes should be paired with revenue‑recycling or investment support in order to preserve cost neutrality.

## 7 Conclusion

Current research on IES that combine wind, solar, gas, and storage has predominantly focused on internal aspects such as technical modeling, economic dispatch, and multi-energy coupling optimization. However, there is a noticeable lack of attention to the external market mechanisms—specifically, how CET and GCT jointly affect the operational performance of IES. This limitation undermines the ability of existing models to support decision-making under increasingly stringent low-carbon policy pressures. Without adequately incorporating carbon costs and renewable energy incentive signals, IES optimization strategies often fail to respond in a timely and effective manner to the demands of carbon neutrality policies, leaving significant carbon reduction potential untapped. Therefore, it is essential to bridge the gap in understanding the interactions between the carbon and green certificate markets within IES operational frameworks, in order to provide scientific and policy-relevant support for system-level decision-making under low-carbon regulatory environments.

### 7.1 Key contributions

This study proposes a two-stage stochastic–robust optimization framework that integrates Monte Carlo-based market forecasting with CVaR risk control. In the first stage, a high-dimensional scenario set is constructed to capture the volatility of carbon prices and green certificate prices. In the second stage, optimization decisions are made under extreme risk constraints [77]. This framework enables the integrated modeling of CET and GCT mechanisms in relation to IES dispatch behavior, offering a systematic solution for low-carbon operational decision-making under complex policy environments. The main theoretical and empirical innovations are as follows:

First, the study achieves a breakthrough academic integration by incorporating CET and GCT within a unified optimization framework, overcoming the conventional separation in previous research. Through coupled modeling and comparative analysis, it reveals the individual and combined effects of the two mechanisms on cost structures, energy mixes, and carbon emission behaviors in IES. This provides both theoretical foundations and empirical evidence for the coordinated optimization of multi-market mechanisms. Results demonstrate that the integrated CET–GCT framework can simultaneously enhance economic efficiency and environmental performance, offering practical insights for incentive structure design by policymakers and system operators [78,79].

Second, the study develops a tri-objective optimization paradigm that systematically integrates affordability, reliability, and sustainability under market volatility. The proposed stochastic decision model extends traditional single-objective energy system optimization by introducing a dynamic multi-criteria equilibrium approach, offering methodological advances for system scheduling and resource allocation in complex energy markets. The price-responsive architecture used in the model is both portable and replicable, establishing a robust computational basis for quantifying the interaction effects of market-based energy policies [80–82].

Third, the study proposes a market mechanism calibration strategy framework, serving as a theoretical tool for redefining the boundaries of market instruments in climate governance. Through scenario analysis, it derives the optimal joint configuration of CET and GCT, achieving a balance between carbon reduction potential and market stability. This offers a transferable governance pathway for aligning and integrating multiple market mechanisms, enriching the institutional design literature in energy policy research.

### 7.2 Practical implications

For policymakers, it is essential to approach the design of CET and GCT from a system-wide perspective, fostering synergy and consistency between the two mechanisms to prevent market failures caused by misaligned incentive signals. This study demonstrates that the coordinated implementation of CET and GCT can significantly enhance both the operational efficiency and emission reduction performance of IES. It is therefore recommended that policy design incorporate the joint optimization of carbon quota allocation, green certificate ratio structures, and trading cycle arrangements. Establishing cross-mechanism pricing signals and incentive boundaries can improve policy stability and predictability, providing a more actionable institutional foundation for achieving dual carbon goals.

For system operators, it is advised to explicitly and systematically integrate carbon costs and green certificate revenues into dispatch and operational decision-making. Developing cross-market price response mechanisms can enhance resource allocation flexibility and improve system resilience. When designing energy pathways and operational strategies, operators should fully recognize the synergistic effects of CET and GCT signals. Investments in energy storage, demand response, and carbon capture should be prioritized to strengthen adaptability to price volatility, enabling proactive management of compliance and cost risks under future tightening of quotas or adjustment of incentive schemes. This approach facilitates a win–win outcome between operational performance and environmental effectiveness.

For investors, attention should be directed toward low-carbon asset portfolios with value premium potential under the CET–GCT interaction regime. Promising investment areas include wind–storage and solar–storage hybrid systems, as well as natural gas with CCUS retrofitting projects, which can deliver stable returns in scenarios of rising carbon prices and enhanced green certificate incentives. It is also recommended to establish carbon and certificate price sensitivity risk assessment mechanisms, treating policy variables as core configuration factors. Forward-looking participation in the construction of a “low-carbon ecosystem”—spanning renewable energy, energy storage, and carbon assets—will enable investors to capture multi-market dividends and seize structural growth opportunities.

### 7.3 Limitations and future research directions

While this study offers significant insights, it also carries certain limitations.

(1) External validity and institutional transferability require systematic testing. The numerical experiments rely on a single Chinese case, and substantial cross-regional heterogeneity in market rules, resource endowments, and load profiles may limit direct generalization. Future studies should apply the framework to multi-regional Chinese datasets and to international contexts to evaluate external validity and to quantify how institutional design shapes operational and environmental performance. When replicating the model in systems such as the EU ETS or California’s cap-and-trade, key institutional parameter sets likely need to be re-specified and calibrated, including cap design and compliance cycles, price containment and risk-mitigation mechanisms, coverage boundaries and offset rules, and the governance of green attributes. Such adaptations would enable robust comparative policy evaluation.
(2) Uncertainty and resilience are simplified and should be strengthened through stochastic–robust decision-making. Wind and solar output and CET and green-certificate prices are treated as deterministic inputs, which does not fully capture renewable intermittency, price volatility, and tail-risk events. Future research could incorporate Monte Carlo scenario sampling, robust or distributionally robust optimization, and budget-of-uncertainty methods. Complementary sensitivity and stress testing under extreme renewable variability and severe weather conditions would provide deeper insight into system resilience under high uncertainty.
(3) Further work is needed on long-term capacity expansion, scalability, and endogenous market feedback. Our core research plan for the next stage is to extend the current day-ahead operational optimization to multi-year capacity expansion planning, which is of profound significance for comprehensively evaluating the impact of CET and GCT on long-term IES investment decisions. Methodologically, nesting macro-level cross-year investment nodes with micro-level hourly operational scheduling introduces multi-time-scale dimensionality and requires characterizing the uncertainty of long-term policy evolution. While this expands the computational footprint, these challenges are readily tractable. We have formulated highly specific countermeasures to address this: we will introduce advanced decomposition algorithms—such as Benders decomposition or Column-and-Constraint Generation (C&CG)—to decouple the framework into a long-term “investment master problem” and a short-term “operational subproblem” for iterative solving. Furthermore, we will deepen the K-medoids clustering technique utilized in this study to extract cross-year “typical weeks/days,” drastically compressing the scenario tree size while preserving the volatility characteristics of renewable energy. To model long-term market dynamics, the existing Monte Carlo price generation mechanism will be expanded into a long-term evolution model incorporating Markov Chains, allowing for the accurate simulation of dynamic CET and GCT price coordination and phased policy shifts over a decade. Crucially, the authors’ established expertise and extensive prior experience in developing large-scale optimization models and decomposition algorithms provide a solid foundation for successfully implementing these methodologies.

Finally, the current formulation assumes price-taking behavior in CET and green-certificate trading. A feasible next step is to model endogenous price formation and cross-market feedback via equilibrium-constrained or bilevel formulations, enabling a richer analysis of competition, policy feedback, and interactions between carbon and green-certificate markets.

## Supporting information

S1 Appendix The input datasets for the Integrated Energy System (IES) model. (XLSX)

S2 Appendix The output datasets for the Integrated Energy System (IES) model. (XLSX)

S3 Appendix The output datasets for the Integrated Energy System (IES) model. (DOCX)
