# Supplementary material for: Optimization of wind-solar-gas-storage integrated energy systems under the carbon and green certificate markets
Source: PLoS One. 2026 Apr 24;21(4):e0346530. doi: 10.1371/journal.pone.0346530 (PMC13108807; doi:10.1371/journal.pone.0346530)
Supplement: S3 Appendix — (DOCX) [file pone.0346530.s003.docx]

**Appendix**

The relevant Nomenclature and Abbreviation in this article are shown in the table below.

**Table A.** Nomenclature and Abbreviation

| integrated energy systems | IES | Renewable Portfolio Standard | RPS |
| --- | --- | --- | --- |
| carbon emission trading | CET | combined heat and power | CHP |
| green certificate trading | GCT | Conditional Value-at-Risk | CVaR |
| European Union Emissions Trading System | EU ETS | energy storage and combined cooling, heating, and power systems | CCHP |
| Guarantees of Origin | GO | volatility of variable renewable energy | VRE |
| Doubly-Fed Induction Generators | DFIGs | neural network-based Direct Power Control | NC-DPC |
| Super-Twisting Algorithm | STA | Sliding Mode Control | SMC |
| Integral Sliding Mode Control | ISMC | Artificial Neural Networks | ANNs |
| Fuzzy Logic Control | FLC | Bald Eagle Search | BES |
| Gradient-Based Optimization | GBO | State of Health | SOH |
| Total free carbon quota of the IES | $C_{L}$ | Carbon trading cost under CET | $C_{{CO}_{2}}$ |
| Carbon quota for gas turbines | $C_{q}$ | Market price of CET | $\lambda$ |
| Carbon quota for gas boilers | $C_{h}$ | Interval width of the CET tiers | $d$ |
| Power output of the gas turbine at time $t$ | $P_{g}^{t}$ | Increment of CET price per tier | $\sigma$ |
| Carbon emission baseline for gas turbines | $B_{g}$ | Cost/revenue from GCT (green certificate trading) | $C_{gre}$ |
| Cooling correction factor for gas turbines (1 for water cooling, 1.05 for air cooling) | $F_{e}$ | Price for buying green certificates | $C_{gre}^{b}$ |
| Heating correction factor, based on the heat-to-power ratio | $F_{r}$ | Price for selling surplus green certificates | $C_{gre}^{s}$ |
| Load (output) correction factor for gas turbines | $F_{f}$ | Actual renewable energy consumption | $P_{w}$ |
| Power output of gas boilers at time $t$ | $Q_{b}^{t}$ | Required minimum renewable energy quota | $P_{res}$ |
| Carbon emission baseline for gas boilers | $B_{h}$ | Penalty coefficient for green certificate shortfall | $C_{f}$ |
| Actual carbon emissions of the IES | $C_{p}$ | Actual output power of wind turbine | $P_{wt}$ |
| Coefficients for carbon emissions from gas turbines | $a_{1},b_{1},c_{1}$ | Rated power of wind turbine | $W_{wt}$ |
| Coefficients for carbon emissions from gas boilers | $a_{2},b_{2},c_{2}$ | Actual wind speed | $\nu$ |
| Number of scheduling intervals (e.g., 24 for day-ahead) | $h$ | Cut-in, rated, and cut-out wind speed | $\upsilon_{in},\upsilon_{r},\upsilon_{out}$ |
| Actual output power of PV module | $P_{p\upsilon}$ | Operating & maintenance cost of thermal storage | $C_{hs}$ |
| Rated power of PV module under STC | $P_{stc}$ | O&M unit cost of thermal storage at time $t$ | $c_{hs}^{t}$ |
| Solar irradiance during project operation | $G_{c}$ | Charging / discharging heat of thermal storage at time $t$ | $Q_{hs,c}^{t},Q_{hs,d}^{t}$ |
| Standard solar irradiance (typically 1000 W/m²) | $G_{stc}$ | Cost of purchasing electricity | $C_{c}$ |
| Power temperature coefficient of PV cells | $\gamma$ | Power purchased from grid at time $t$ | $P_{grid}\left( t \right)$ |
| Actual temperature | $T_{c}$ | Electricity price at time $t$ | $C_{grid}\left( t \right)$ |
| Standard temperature | $T_{stc}$ | Duration of each scheduling period | $\Delta t$ |
| Power output of power generation unit (PGU) | $P_{PGU}$ | Cost of purchasing gas | $C_{gas}$ |
| Heat input to PGU | $Q_{PGU}$ | Unit price of gas | $c_{gas}$ |
| Power generation efficiency of PGU | $\eta_{e}$ | Input power to the absorption chiller | $P_{AC}$ |
| Rated output power of PGU | $P_{PGU,max}$ | Output power of the gas boiler | $P_{GB}$ |
| Operation status of PGU at time $t$: 0 for off, 1 for on | $U_{PGU}\left( t \right)$ | Output power of absorption chiller and electric chiller respectively | $P_{AC}^{*},P_{EC}^{*}$ |
| State of charge of battery at time $t$ | $SOC\left( t \right)$ | Thermal and cooling load demand of IES | $Q_{h,load},Q_{c,load}$ |
| Charging power of battery storage at time $t$ | $P_{BS}^{ch}\left( t \right)$ | Minimum selling price of green certificates | $C_{gre}^{min}$ |
| Discharging power of battery storage at time $t$ | $P_{BS}^{dis}\left( t \right)$ | Maximum selling price of green certificates | $C_{gre}^{max}$ |
| Charging / discharging efficiency of battery | $\eta_{BS}^{ch}\left( t \right),\eta_{BS}^{dis}\left( t \right)$ | On-grid electricity price for type‑i green certificate | $s_{i}$ |
| Rated capacity of battery | $C_{bat}$ | Benchmark electricity price of gas turbine | $c$ |
| Purchase cost of battery | $C_{purchase}$ | Discount rate for type‑i green certificate | $r_{i}$ |
| Number of charge-discharge cycles of battery | $M$ | Subsidy settlement cycle for type‑i certificate | $h_{i}$ |
| Amortized cost per cycle | $C_{r}$ | Deferred subsidy payment period for type‑i certificate | $d_{i}$ |
| Operating & maintenance cost of battery | $C_{es}$ | Proportion of renewable energy generation by GCT party in i-th category | $\alpha_{i}$ |

The complete pseudocode of this research framework covers four major steps: policy mechanism analysis, system modeling, optimization modeling, numerical solution, and result analysis, as follows:

**Table B.** Pseudocode of Research Framework

| Optimization of Integrated Energy Systems under Joint Carbon Emission Trading (CET) and Green Certificate (GCT) Mechanisms. |
| --- |
| # Step 0: Initialize system parameters and market mechanisms  Input:  T ← total time periods (e.g., 24 hours)  Δt ← time step (e.g., 1 hour)  Equipment params ← parameters for PV, wind turbine, CHP, boiler, battery, etc.  Load profile ← hourly electric, heating, and cooling demand  Price profile ← electricity price, natural gas price, CET & GCT prices  CET mechanism ← {free quotas, tiered pricing structure}  GCT mechanism ← {RPS quotas, price bounds, penalties}  # Step 1: Scenario generation and reduction  Generate N Monte Carlo scenarios for: wind speed, solar irradiance, electricity price, CET and GCT price trajectories  Apply k-medoids clustering to reduce scenario tree:  Scenario_Set ← representative scenarios for optimization  # Step 2: IES system modeling  For each device $e$ $\in${PV, WT, CHP, Boiler, Battery, Thermal Storage}:  Define power output model  For each time t ∈ [1, T]:  Add energy balance constraints: Electrical balance, Thermal balance, Cooling balance  Model battery and thermal storage SOC:  # Step 3: MILP-based optimization model  Define decision variables  Objective:  Minimize Total Cost  Subject to:  Power/thermal/cooling balance constraints  Capacity and efficiency limits of all devices  SOC upper/lower bounds  CET pricing model: tiered emission costs  GCT constraints: quota satisfaction, certificate price limits, penalties  Cross-checking CET and GCT credits to avoid double counting  # Step 4: MILP solving for each scenario  For scenario in Scenario Set:  Solve MILP via YALMIP + CPLEX  Store optimal scheduling results and costs  # Step 5: Post-processing and evaluation  For each scenario:  Output dispatch strategy for all equipment  Decompose total cost (electricity, gas, CET, GCT)  Analyze emission reduction and quota usage  Evaluate sensitivity to CET/GCT pricing and policy structure  Output:  Optimal IES operation strategy  Economic and environmental performance  Policy effectiveness under CET-GCT interaction |
